# Abundance and diversity of gut-symbiotic bacteria, the genus *Burkholderia* in overwintering *Riptortus pedestris* (Hemiptera: Alydidae) populations and soil in South Korea

**DOI:** 10.1371/journal.pone.0218240

**Published:** 2019-06-13

**Authors:** Minhyung Jung, Doo-Hyung Lee

**Affiliations:** Department of Life Sciences, Gachon University, Seongnam-si, Gyeonggi-do, South Korea; Northwest A&F University, CHINA

## Abstract

*Riptortus pedestris* is a major agricultural pest on leguminous plants in South Korea and Japan. Recent studies have revealed that *R*. *pedestris* can form beneficial symbiosis with bacteria belonging to genus *Burkholderia* acquired from soil newly for every generation. Although their physiological interactions are relatively well-understood, infection rate and abundance of the *Burkholderia* in overwintering natural populations of *R*. *pedestris* remain unknown. Therefore, the objective of this study was to characterize *Burkholderia* infection ratio and clade composition of overwintering *R*. *pedestris* populations as well as prevalence and diversity of the genus *Burkholderia* in soil by conducting a two-year field survey. From the field survey, we found 29 overwintering *R*. *pedestris* adults in forested areas nearby soybean fields. Diagnostic PCR analysis revealed that overall infection rate of the symbiotic *Burkholderia* was 93.1% from overwintering adults. Among the *Burkholderia*-infected *R*. *pedestris*, 70.4% of individuals harbored unclassified *Burkholderia* clades whereas 22.2% and 7.4% of *R*. *pedestris* harbor stinkbug-associated beneficial and environmental (SBE) group and *Burkholderia cepacia* and complex (BCC), respectively. All *R*. *pedestris* were infected with a single clade of *Burkholderia*. In soil, 56.2% of soil samples were *Burkholderia* positive, and unlike *R*. *pedestris*, multiple *Burkholderia* clades were detected from 62.2% of those samples. Clade composition of the genus *Burkholderia* in the samples with the bacteria was 91.1%, 60.0%, 31.1% and 8.8% for plant-associated beneficial and environment (PBE), BCC, SBE and unclassified clade, respectively.

## Introduction

During winter season, insects face thermally stressful environments. They have developed various strategies to successfully withstand and survive inhospitable environments through evolutionary interactions with organisms and abiotic conditions [1,2]. Their physiological strategies for cold hardness can be classified into two groups: freeze tolerant (synthesis of ice nucleating agents) and freeze avoidance (removal of all potential nucleates) [2–5]. In addition, insects can behaviorally avoid inhospitable environments through long or short distance movement to less stressful conditions [2,6,7].

Understanding overwintering ecology is particularly important for pest species because levels of survived winter populations can serve as a barometer for estimating pest pressures in succeeding active seasons [1,3,4]. Indeed, there have been attempts to better manage pest populations by estimating overwintering population levels. For examples, the timing and size of spring emerge populations have been forecasted for aphid management by evaluating overwintering eggs of black bean aphid, *Aphis fabae* Scolpoli (Hemiptera: Aphididae) [1,8,9] and cereal aphid, *Rhopalosiphum padi* Linnaeus (Hemiptera: Aphididae) [10].

Although overall density levels of overwintering pest populations are undoubtedly foundational information to forecast pest pressures in the spring, biological variations at individual level are also worthwhile to consider because these variances could affect the survival rate of overwintering insects themselves as well as their performance after emergence [1,11]. Physiological status and fitness of overwintering individuals are affected by various abiotic and biotic factors. Among them, symbiotic associations with microorganisms can play a pivotal role by making arthropod hosts more tolerant to thermally stressful environments [12–15]. Furthermore, it has been reported that symbiotic microbes can also enhance individual fitness of host arthropods under cold conditions [16].

The bean bug, *Riptortus pedestris* (Fabricius) (Hemiptera: Alydidae), is a polyphagous insect pest in East Asia including South Korea, Japan, and Taiwan [17–19]. Both adult and immature stages of *R*. *pedestris* attack leguminous plants, especially soybeans [17,20–22]. Recently, it has been revealed that *R*. *pedestris* possess symbiotic bacteria of the genus *Burkholderia* in the midgut crypts [23,24]. Interestingly, unlike typical insect-microbe symbiosis, *Burkholderia* are not transmitted vertically from parents to offspring. Instead, *R*. *pedestris* acquire their symbionts newly at every generation from soil environments mainly during the 2nd instar period [24–26]. Forming an insect-microbe symbiosis, symbionts are known to provide a series of enhancement of host fitness, including increased fecundity, decreased developmental period, and increased body size [24,25,27].

Although the effect of *Burkholderia* infection on the biology of active, non-diapausing *R*. *pedestris* is relatively well understood, interaction between symbionts and overwintering *R*. *pedestris* remains unknown. It is likely that *R*. *pedestris* with *Burkholderia* would impose a higher risk level against pest management compared to aposymbiotic individuals because symbiotic individuals are known to exhibit enhanced life history parameters such as fecundity and developmental rate. Moreover, Kikuchi et al. [28] have found that when *R*. *pedestris* acquire fenitrothion-degrading *Burkholderia* strain, these insects can have resistance to fenitrothion, a widely used insecticide against *R*. *pedestris*. Therefore, the infection rate of *Burkholderia* in overwintering *R*. *pedestris* population could serve as a basic information to accurately assess the risk level of overwintering population to crops in the spring.

*Riptortus pedestris* are known to enter facultative diapause, as opposed to preprogramed obligate diapause, in adult stage with response to abiotic changes especially for short-day photoperiods [29–31]. In addition, a recent study has revealed that adult *R*. *pedestris* can use leaf litter as a primary overwintering structure and that overwintering individuals are randomly distributed when forested areas adjacent to crop fields are surveyed [32]. The objective of the present study was to determine infection rate and clade composition of *Burkholderia* in overwintering *R*. *pedestris* and prevalence and diversity of the genus *Burkholderia* in soil environments by conducting a two-year field survey in South Korea. We collected leaf litter from forested areas nearby soybean fields from eight provinces in South Korea to locate overwintering *R*. *pedestris*. We also collected soil samples at the same locations from which leaf litter were collected. All overwintering *R*. *pedestris* found and soil samples collected were individually subjected to molecular analysis to assess the prevalence of *Burkholderia* in both insects and soil samples.

## Materials and methods

### Field survey

**Fig 1:**
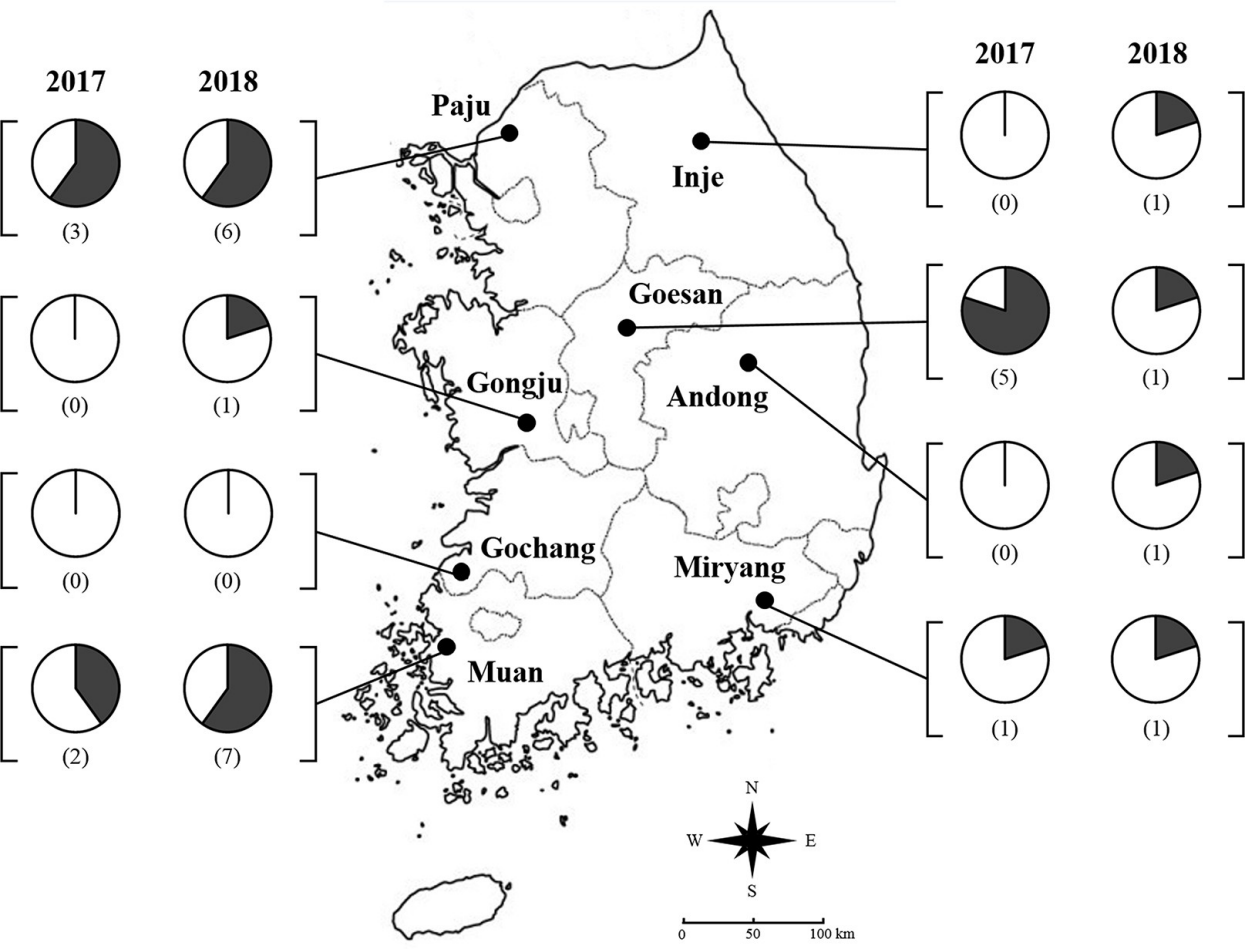
Geographical locations of eight sampling sites in South Korea from the two-year field survey. Pie chart indicate the ratio of leaf litter samples in which overwintering *Riptortus pedestris* found from 2017 and 2018, respectively. Parentheses present below the pie chart show the number of overwintering *R*. *pedestris* found in leaf litter samples collected.

A field survey was conducted over two winter seasons from January to February in 2017 and 2018 to characterize infection rate and clade composition of genus *Burkholderia* in overwintering *R*. *pedestris* populations as well as prevalence and diversity of *Burkholderia* in soil samples of South Korea. For the survey, a representative soybean field was selected in each province in South Korea based on the Korean Statistical Information Service (KOSIS) [33]: Paju (37°57'22.06"N 126°52'7.53"E), Inje (37°57'57.61"N 128°17'35.09"E), Goesan (36°53'36.91"N 127°49'22.61"E), Gongju (36°17'3.14"N 127° 2'25.65"E), Andong (36°36'45.12"N 128°37'1.22"E), Miryang (35°23'6.74"N 128°47'7.99"E), Gochang (35°25'50.50"N 126°37'37.46"E), and Muan (34°59'43.24"N 126°27'21.62"E) (Fig 1). In each province, a sampling site was selected in the forested area located within ca. 1 km from the soybean field. In each sampling site, five sampling points were established with a distance of ca. 20 m apart from each other located at similar elevations. At each sampling point, a 1 × 1 m quadrat was drawn. Then all organic materials over the soil layer mainly consisting of leaf litter were carefully collected into a plastic bag [34]. After collecting the leaf litter sample, 30 ml of soil sample was also collected and sealed in a 50 ml conical tube (Hyundai micro Co., Ltd., Seoul, South Korea).

### Sample processing

Collected leaf litter samples were brought to the laboratory and kept in an indoor area (temperature ca. 4°C) until samples were examined to locate overwintering *R*. *pedestris*. When live overwintering *R*. *pedestris* or other live overwintering hemipteran species were detected, insects were individually kept in 1.5-ml conical tubes (Axygen Biosciences, Hangzhou, China) with 90% ethanol after species and sex identification. Dominant leaf types in sampling areas were confirmed based on Forest Geospatial Information System (FGIS) [34]. After sample inspection, leaf litter samples were completely dried in a dry-oven (BF-150C, Biofree Co. Ltd., Seoul, South Korea) at 80°C for 72 hours to estimate moisture contents.

### DNA extraction

Collected overwintering *R*. *pedestris* and soil samples were individually subjected to DNA extraction and diagnostic PCR. The abdomen part of *R*. *pedestris* was used for DNA extraction using MagListo^TM^ 5M Genomic DNA Extraction Kit (Bioneer Co. Ltd., Daejeon, South Korea) according to the manufacturer’s instructions. DNA extractions for soil samples were performed with 25 mg of soil per sampling point using DNeasy PowerSoil kit (QLAGEN, Hilden, Germany).

### Diagnostic PCR

PCR analysis was conducted to determine the presence of specific *Burkholderia* clades in total DNA extracts from insect and soil samples. First, all samples were subjected to PCR analysis to detect the presence of genus *Burkholderia* using *Burkholderia*-specific 16s rRNA primers (Table 1).

**Table 1:** Primers and probes used in this study.

Target group	Target gene	Primer/Probe name	Nucleotide sequence (5’ → 3’)	Approximate product size (kb)	Annealing temp (°C)	Reference
*Burkholderia*						
*Burkholderia* spp.	16s rRNA	Burk16SF	TTTTGGACAATGGGGGCAAC	0.7	55	Kikuchi et al. [23]
		Burk16SR	GCTCTTGCGTAGCAACTAAG			
PBE clade	16S rRNA	Burk16SF	TTTTGGACAATGGGGGCAAC	0.5	55	Itoh et al. [35]
		PBE822R	CTTCGTTACCAAGTCAATGAAGA			
BCC clade	16S rRNA	BCC370F	TTTTGGACAATGGGCGAAAG	0.8	55	Itoh et al. [35]
		Burk16SR	GCTCTTGCGTAGCAACTAAG			
SBE clade	16S rRNA	SBE160F	CGCATACGACCTAAGGGA	1.3	55	Itoh et al. [35]
		SBE1400R	CTTGCGGTTAGGCTACCT			
*R*. *pedestris*	COI	LCO1490	GGTCAACAAATCATAAAGATATTGG	0.7	48	Folmer et al. [36]
		HCO2198	TAAACTTCAGGGTGACCAAAAAATCA			

Then, *Burkholderia* positive samples were subjected to subsequent PCR analysis to determine the *Burkholderia* clade composition in the samples using clade-specific primer sets including BCC, PBE, and SBE clades (Table 1) with temperature profile of 94°C for 5 min followed by 35 cycles of 94°C for 30s, 55°C for 1 min, and 72°C for 2 min [26,35]. Gel electrophoresis was performed to confirm the presence of target primers on a 1.0% agarose gel slab containing Tris-acetate-EDTA buffer (40 mM Tris-Acetate, 1 mM EDTA, pH 8.3) at 120 v/cm (Mupid-exU, Takara Bio Inc., Shiga, Japan) followed by visualization on a digital gel documentation system. *Burkholderia* positive samples that did not belong to any of the three clades were defined as unclassified in this study.

## Results

### Overwintering *R*. *pedestris* populations

From the two-year field survey, a total of 11 and 18 overwintering *R*. *pedestris* adults were collected from 2017 and 2018, respectively (Fig 1 and S1 Table). Among the eight provinces surveyed, no overwintering *R*. *pedestris* was found in Gochang over the two-year survey whereas overwintering individuals were found from Paju, Muan, Goesan, and Miryang in both years. The greatest number (a total of 9 individuals found per province) of overwintering *R*. *pedestris* was found in Paju and Muan over two years. Percentages of leaf litter samples containing *R*. *pedestris* ranged from 0 to 80% in both 2017 and 2018 across provinces surveyed. In general, overwintering *R*. *pedestris* were present solitarily in leaf litter samples (76.2% of leaf litters contained a single individual). Over the two-year survey, a total of 12 males and 17 females were found. The sex ratio was not significantly deviated from 1:1 (Likelihood Ratio = 0.4415, *P* = 0.5061). In addition, a total of 22 hemipteran species in families of Coreidae and Pentatomidae were collected over two years, including *Cletus punctiger* Dallas (Hemiptera: Coreidae), winter cherry bug, *Acanthocoris sordidus* Thunberg (Hemiptera: Coreidae), and brown marmorated stink bug, *Halyomorpha halys* Stål (Hemiptera: Pentatomidae).

**Fig 2:**
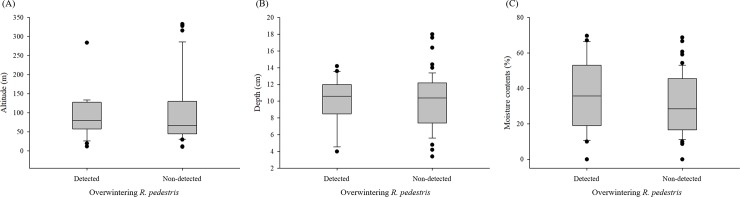
Characteristics of leaf litter samples with or without overwintering *Riptortus pedestris* found. (A) Altitude of leaf litter location, (B) Depth of leaf litter sample above the soil base layer, and (C) Moisture content of leaf litter sample.

Characteristics of leaf litter samples were compared between the two groups with and without overwintering *R*. *pedestris* found (Fig 2). There was no significant difference in the altitude of leaf litter location between the two groups (*t* = 0.75, *P* = 0.4556). There was no significant difference in depth (*t* = 0.20, *P* = 0.8348) or moisture content (*t* = 0.94, *P* = 0.3512) between *R*. *pedestris*-detected and non-detected samples collected either. Based on FGIS [34], Overwintering *R*. *pedestris* were almost exclusively detected from deciduous leaf leaves especially from Family Fagaceae whereas only one individual was found from a forest mainly with Family Pinaceae.

### Characteristics of *Burkholderia* in overwintering *R*. *pedestris*

**Fig 3:**
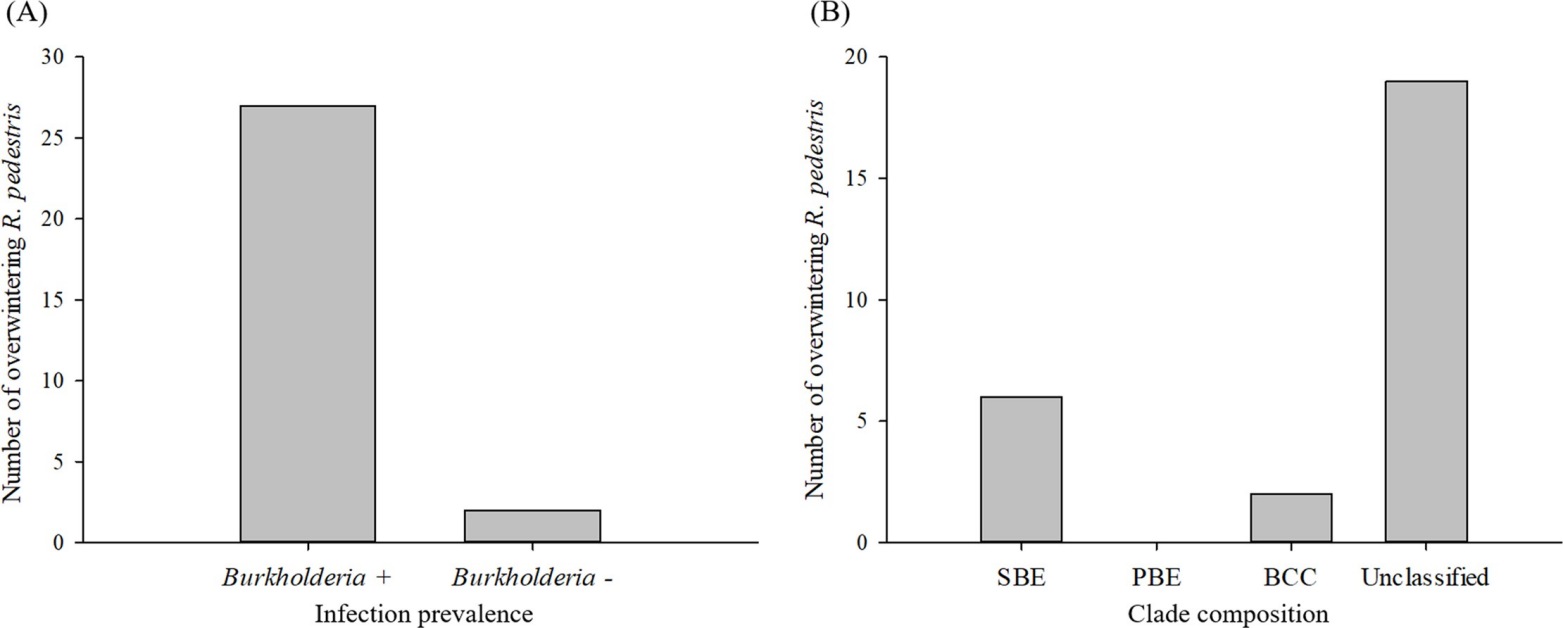
Characteristics of *Burkholderia* infection and clade composition in overwintering *Riptortus pedestris* found from leaf litter samples over the two-year survey. (A) Overall number of insects infected with genus *Burkholderia*, and (B) Clade composition of genus *Burkholderia* in insects.

Among 29 overwintering *R*. *pedestris* collected over two years, 93.1% of individuals were infected with genus *Burkholderia* (S1 Table). Only two individuals found in 2018 were not infected with these bacteria (Fig 3). In *Burkholderia*-infected *R*. *pedestris*, 70.3% of individuals harbored unclassified *Burkholderia* strains whereas 22.2 and 7.4% of *Burkholderia* found in *R*. *pedestris* were identified as SBE and BCC clades, respectively (Fig 3). All infected *R*. *pedestris* individuals harbored a single *Burkholderia* clade without multiple strain infection.

### Characteristics of *Burkholderia* in soil

**Fig 4:**
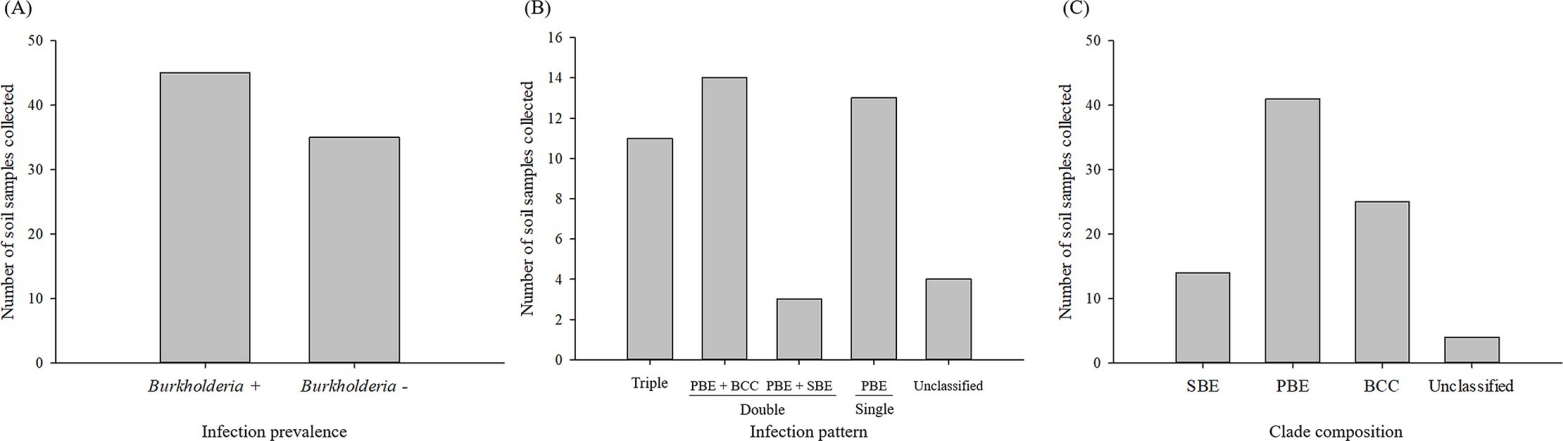
Characteristics of *Burkholderia* positive and clade composition in soil samples collected over the two-year survey. (A)Overall number of genus *Burkholderia* positive soil, (B) Clade composition of genus *Burkholderia* in the soil, and (C) Total frequency of *Burkholderia* clade detected from soil samples.

Among 80 soil samples collected over two years, 56.2% of soil samples were *Burkholderia* positive (Fig 4 and S2 Table). In *Burkholderia* positive soil samples, 24.4% of soil samples were detected with all three clades in genus *Burkholderia* tested, whereas 31.1 and 6.7% of samples were detected with PBE + BCC clades and PBE + SBE clades, respectively (Fig 4). In addition, 28.9% samples were detected only with a single clade, PBE, while 8.9% samples were positive for unclassified *Burkholderia*. Overall, PBE clade was most frequently detected from soil samples. This clade was detected in 41 out of 55 *Burkholderia* positive soil samples (Fig 4).

### Ethics statement

No specific permits were required for the described field survey.

## Discussion

Infection rate and clade composition of symbiotic *Burkholderia* in *R*. *pedestris* field populations provides critical information to better design and implement pest management programs against this economically-important pest. Although it is currently unknown how symbionts affect the biology of overwintering *R*. *pedestris*, it is expected that higher infection rates in overwintering populations would impose higher risk to crops by survived pests in the spring. This is because symbiotic *Burkholderia* are known to provide a series of enhancement for *R*. *pedestris* fitness, including larger body size, increased fecundity, and enhanced insecticide resistance [24,25,28]. For this reason, this study investigated infection rate of *Burkholderia* in overwintering *R*. *pedestris* populations in nature for the first time. In this study, DNA sample was extracted from the whole abdomen of overwintering *R*. *pedestris*, which might have resulted in detection of *Burkholderia* from gut sections other than the crypt-bearing M4 region, where the symbiont is known to colonize in the insect. Nevertheless, overwintering individuals generally clear their gut lumen to prevent freeze damage because the particles in gut lumen can act as ice nucleators, reducing the chance of detecting the symbionts from other gut sections [2].

Results of this study indicate that overwintering adults of *R*. *pedestris* are infected with *Burkholderia* with a high rate, yielding 93.1% across the two-year survey. This high infection rate in overwintering adults is consistent with results of field study conducted by Kikuchi et al. [23] in Japan. They examined active, non-diapausing *R*. *pedestris* adults and found that the infection rate was as high as 97.8%. However, we should carefully interpret the high infection rate in overwintering populations because it is currently unknown whether *Burkholderia* infection affects searching behavior of *R*. *pedestris* adults for overwintering sites or their survivorship during the winter. That is, we cannot rule out the possibility that aposymbiotic individuals are less likely to successfully locate overwintering sites or may suffer from higher mortality in thermally stressful environments. Indeed, a recent study has revealed that a specific bacteria species in *Drosophila* microbiome is essential for the fly to exhibit coordinated locomotion behavior [37]. Moreover, the survival rate and mobility of black-legged tick are significantly enhanced when they form symbiosis with *Anaplasma phagocyophilum* [16].

With respect to clade composition of *Burkholderia* found in overwintering *R*. *pedestris*, SBE and BCC clades were detected from the abdomen of this insect. The infection rate with SBE clade known to specifically form associations with stink bugs [24] was 22.2% among overwintering individuals. Therefore, these insects with SBE might have enhanced fitness such as fecundity as reported in previous studies [24,25]. Interestingly, two overwintering *R*. *pedestris* harbored BCC clade in their body. BCC clade is known as a pathogen infecting human, animals, and plants without previous report of its association with *R*. *pedestris*. This result suggests that relationships of BCC clade with *R*. *pedestris* and potential effects of the clade on the biology of insects need to be investigated in the future. In addition, 70.3% of *Burkholderia* found in the overwintering *R*. *pedestris* were not phylogenetically grouped into any of the three clades previously classified. Thus, further studies should identify phylogenetic relationships of this unclassified *Burkholderia* group with other clades and its biological associations with *R*. *pedestris*.

At geographical scale of this study, the percentage of *Burkholderia* positive soil samples was substantially lower than that found from overwintering *R*. *pedestris*. Moreover, SBE clade was detected from only 17.5% of the samples. As somewhat expected, PBE clade known as a plant-associated beneficial and environmental group that could promote plant growth [24,38], was the most prevalent group in soil, with PBE clade being detected from 51.3% of the soil samples. Although the proportion of *Burkholderia* positive samples in soil was slightly greater than 50%, it was notable that the soil samples detected with multiple *Burkholderia* clades were fairly common in *Burkholderia*-positive samples. These results indicate that *Burkholderia* are likely to be distributed in patch among soil environments, although multiple clades are present together in the patch. Nevertheless, soil microbiota tends to be complex with enormously diverse microorganisms and therefore our use of diagnostic PCR could have resulted in false-positives. For this reason, caution is needed when interpreting the soil sample results. Further analysis using sequencing or next generation sequencing may be warranted to identify and characterize their composition at a higher accuracy.

Our results revealed that all *R*. *pedestris* harboring *Burkholderia* were infected with a single clade. This strongly suggests that *R*. *pedestris* insects are capable of selectively colonizing their mid-gut with a single, specific *Burkholderia* clade. Indeed, Ohbayashi et al. [39] have demonstrated that *R*. *pedestris* insects have an intestinal organ known as constricted region to sort symbionts for selective colonization by *Burkholderia* in the mid-gut.

Consistent with results of a previous study [32], our two-year survey confirmed that adult *R*. *pedestris* could overwinter in the forested area adjacent to crop fields. This study also revealed that this insect species could utilize deciduous leaf litter in family Fagaceae as overwintering structures. However, there was no evidence supporting that the altitude of leaf litter location, the depth of leaf litter, or the moisture content of leaf litter could affect the likelihood of finding overwintering individuals. That is, it is not feasible with current results to recommend site-specific management tactics within the landscape scale of this study. Further studies should examine variations of micro climate conditions within deciduous forest and their potential effects on overwintering site selection by *R*. *pedestris*.

In summary, we characterized the infection rate and clade composition of *Burkholderia* in overwintering insect populations for the first time. In addition, we report the prevalence and clade composition of *Burkholderia* in soil which serves as a primary environment for *R*. *pedestris* to newly acquire symbionts for every generation. In most pest management systems, overwintering ecology of target species has been neglected due to difficulty of field survey [1]. Therefore, information reported here may serve as basis to improve pest management programs against *R*. *pedestris* by characterizing risk levels of pest populations based on the size of overwintering populations and their symbiont-associated fitness levels.

## Supporting information

S1 Table Infection prevalence and clade composition of the genus *Burkholderia* in the overwintering *R*. *pedestris* detected over 2-yr field survey. (DOCX)

S2 Table *Burkholderia* positive ratio and clade composition in soil samples collected over the two-year survey. (DOCX)

## References

[1] Leather SR, Walters KFA, Bale JS. The ecology of insect overwintering Cambridge University Press, Cambridge, UK; 1993.

[2] Bale JS, Hayward SAL. Insect overwintering in a changing climate. J Exp Biol. 2010; 213: 980–994. 10.1242/jeb.037911 20190123

[3] Salt RW. Principles of insect cold-hardiness. Annu Rev Entomol. 1961; 6: 55–74.

[4] Danks HV. Insect adaptations to cold and changing environments. Can Entomol. 2006; 138: 1–23.

[5] Danks HV. Winter habitats and ecological adaptations for winter survival In: Insects at low temperature. Springer, Boston, MA, 1991; pp. 231–259.

[6] Kennedy GG, Storer NP. Life systems of polyphagous arthropod pests in temporally unstable cropping systems. Annu Rev Entomol. 2000; 45: 467–493. 10.1146/annurev.ento.45.1.467 10761586

[7] Lombaert E, Boll R, Lapchin L. Dispersal strategies of phytophagous insects at a local scale: adaptive potential of aphids in an agricultural environment. BMC Evol Biol. 2006; 6: 75 10.1186/1471-2148-6-75 17014710 PMC1622755

[8] Way MJ, Cammell ME, Taylor LR, Woiwod IP. The use of egg counts and suction trap samples to forecast the infestation of spring‐sown field beans, *Vicia faba*, by the black bean aphid, *Aphis fabae*. Ann Appl Biol. 1981; 98: 21–34.

[9] Leather SR. Forecasting aphid outbreaks using winter egg counts: An assessment of its feasibility and an example of its application in Finland. J Appl Entomol. 1983; 96: 282–287.

[10] Klueken AM, Hau B, Ulber B, Poehling HM. Forecasting migration of cereal aphids (Hemiptera: Aphididae) in autumn and spring. J Appl Entomol. 2009; 133: 328–344.

[11] Gullan PJ, Cranston PS. The Insects: An Outline of Entomology. 4th ed John Willey & Sons, Chichester, UK; 2010.

[12] Engel P, Moran NA. The gut microbiota of insects–diversity in structure and function. FEMS Microbiol Rev. 2013; 37: 699–735. 10.1111/1574-6976.12025 23692388

[13] Feldhaar H. Bacterial symbionts as mediators of ecologically important traits of insect hosts. Ecol Entomol. 2011; 36: 533–543.

[14] Russell JA, Moran NA. Costs and benefits of symbiont infection in aphids: variation among symbionts and across temperatures. Proc R Soc Lond [Biol]. 2005; 273: 603–610. 10.1098/rspb.2005.3348 PMC1560055 16537132

[15] Brumin M, Kontsedalov S, Ghanim M. *Rickettsia* influences thermotolerance in the whitefly *Bemisia tabaci* B biotype. Insect Sci. 2011; 18: 57–66.

[16] Neelakanta G, Sultana H, Fish D, Anderson JF, Fikrig E. *Anaplasma phagocytophilum* induces *Ixodes scapularis* ticks to express an antifreeze glycoprotein gene that enhances their survival in the cold. J Clin Invest. 2010; 120: 3179–3190 10.1172/JCI42868 20739755 PMC2929727

[17] Kono S. Number of annual generations of the bean bug, *Riptortus clavatus* Thunberg (Heteroptera: Alydidae) estimated by physiological characteristic. Jap J Appl Entomol Zool. 1989; 33: 128–133.

[18] Kikuhara Y. The Japanese species of the genus Riptortus (Heteroptera, Alydidae) with description of a new species. Jpn J Syst Entomol. 2005; 11: 299–311.

[19] Lim UT. Occurrence and control method of *Riptortus pedestris* (Hemiptera: Alydidae): Korean perspectives. Korean J Appl Entomol. 2013; 52: 437–448.

[20] Son C, Park S, Hwang Y, Choi B. Field occurrence of stink bug and its damage in soybean. Korean J Crop Sci. 2000; 45: 405–410.

[21] Kang CH, Huh HS, Park CG. Review on true bugs infesting tree fruits, upland crops, and weeds in Korea. Korean J Appl Entomol. 2003; 42: 269–277.

[22] Lee GH, Paik CH, Choi MY, Oh YJ, Kim DH, Na SY. Seasonal occurrence, soybean damage and control efficacy of bean bug, *Riptortus clavatus* Thunberg (Hemiptera: Alydidae) at soybean field in Honam province. Korean J Appl Entomol. 2004; 43: 249–255.

[23] Kikuchi Y, Meng XY, Fukatsu T. Gut symbiotic bacteria of the genus *Burkholderia* in the broad-headed bugs *Riptortus clavatus* and *Leptocorisa chinensis* (Heteroptera: Alydidae). Appl Environ Microbiol. 2005; 71: 4035–4043. 10.1128/AEM.71.7.4035-4043.2005 16000818 PMC1169019

[24] Takeshita K, Kikuchi Y. *Riptortus pedestris* and *Burkholderia* symbiont: an ideal model system for insect–microbe symbiotic associations. Res Microbial. 2017; 168: 175–187. 10.1016/j.resmic.2016.11.005 27965151

[25] Kikuchi Y, Hosokawa T, Fukatsu T. Insect-microbe mutualism without vertical transmission: a stinkbug acquires a beneficial gut symbiont from the environment every generation. Appl Environ Microbiol. 2007; 73:4308–4316. 10.1128/AEM.00067-07 17483286 PMC1932760

[26] Kikuchi Y, Hosokawa T, Fukatsu T. Specific developmental window for establishment of an insect-microbe gut symbiosis. Appl Environ Microbiol. 2011; 77: 4075–4081. 10.1128/AEM.00358-11 21531836 PMC3131632

[27] Kikuchi Y, Fukatsu T. Live imaging of symbiosis: spatiotemporal infection dynamics of a GFP‐labelled *Burkholderia* symbiont in the bean bug *Riptortus pedestris*. Mol Ecol. 2014; 23: 1445–1456. 10.1111/mec.12479 24103110 PMC4238818

[28] Kikuchi Y, Hayatsu M, Hosokawa T, Nagayama A, Tago K, Fukatsu T. Symbiont-mediated insecticide resistance. Proc Natl Acad Sci U.S.A. 2012; 109: 8618–8622. 10.1073/pnas.1200231109 22529384 PMC3365206

[29] Numata H, Hidaka T. Photoperiodic control of adult diapause in the bean bug, *Riptortus clavatus* Thunberg (Heteroptera: Coreidae) I. Reversible induction and termination of diapause. Jap J Appl Entomol Zool. 1982; 17: 530–538.

[30] Numata H, Hidaka T. Photoperiodic control of adult diapause in the bean bug, *Riptortus clavatus* Thunberg (Heteroptera: Coreidae) II. Termination of diapause induced under different photoperiods. Jap J Appl Entomol Zool. 1983; 18: 439–441.

[31] Numata H, Hidaka T. Photoperiodic control of adult diapause in the bean bug, *Riptortus clavatus* Thunberg (Heteroptera: Coreidae) IV.: Food and post-diapause development. Jap J Appl Entomol Zool. 1984; 19: 443–447.

[32] Jung M, Lee DH. Characterization of Overwintering Behaviors and Sites of Bean Bug, *Riptortus pedestris* (Hemiptera: Alydidae), Under Laboratory and Field Conditions. Environ Entomol. 2018; 47: 1280–1286. 10.1093/ee/nvy123 30137287

[33] KOSIS (Korea Statistical Information Service). Statistics of national soybean production in South Korea. Korea Statistical Information Service, Daejeon, South Korea, http://www.kosis.kr.

[34] FGIS (Forest Geospatial Information System). Tree identification map of South Korea. Korea Forest Service, Daejeon, South Korea, http://map.forest.go.kr.

[35] Itoh H, Aita M, Nagayama A, Meng XY, Kamagata Y, Navarro R, Hori T, Ohgiya S, Kikuchi Y. Evidence of environmental and vertical transmission of *Burkholderia* symbionts in the oriental chinch bug *Cavelerius saccharivorus* (Heteroptera: Blissidae). Appl Environ Microbiol. 2014; 80: 5794–5983. 10.1128/AEM.01087-14 PMC4178689 25038101

[36] Folmer O, Black M, Hoeh W, Lutz R, Vrijenhoek RC. DNA primers for amplification of mitochondrial cytochrome C oxidase subunit I from metazoan invertebrates. Mol Mar Biol Biotechnol. 2014; 3: 294–299. 7881515

[37] Schretter CE, Vielmetter J, Bartos I, Marka Z, Marka S, Argade S, Mazmanian SK. A gut microbial factor modulates locomotor behaviour in Drosophila. Nature. 2018; 563: 402 10.1038/s41586-018-0634-9 30356215 PMC6237646

[38] Compant S, Nowak J, Coenye T, Clément C, Ait Barka E. Diversity and occurrence of *Burkholderia* spp. in the natural environment. FEMS Microbial Rev. 2008; 32: 607–626. 10.1111/j.1574-6976.2008.00113.x 18422616

[39] Ohbayashi T, Takeshita K, Kitagawa W, Nikoh N, Koga R, Meng XY et al Insect’s intestinal organ for symbiont sorting. Proc Natl Acad Sci U.S.A. 2015;112: E5179–E5188. 10.1073/pnas.1511454112 26324935 PMC4577176

