# Supplementary material for: Abundance and diversity of gut-symbiotic bacteria, the genus Burkholderia in overwintering Riptortus pedestris (Hemiptera: Alydidae) populations and soil in South Korea
Source: PLoS One. 2019 Jun 13;14(6):e0218240. doi: 10.1371/journal.pone.0218240 (PMC6563995; doi:10.1371/journal.pone.0218240)
Supplement: S1 Table — (DOCX) [file pone.0218240.s001.docx]

| Year | Site | ID | Sex | *Burkholderia*  infection | Clade composition | | | |
| --- | --- | --- | --- | --- | --- | --- | --- | --- |
|  |  |  |  |  | PBE | BCC&P | SBE | Unclassified |
| 2017 | Paju | 17P1A | Male | + |  | ○ |  |  |
| 2017 | Paju | 17P1B | Female | + |  |  |  | ○ |
| 2017 | Paju | 17P2A | Female | + |  |  |  | ○ |
| 2017 | Goesan | 17G1A | Female | + |  |  |  | ○ |
| 2017 | Goesan | 17G3A | Female | + |  |  |  | ○ |
| 2017 | Goesan | 17G3B | Female | + |  |  | ○ |  |
| 2017 | Goesan | 17G4A | Male | + |  |  |  | ○ |
| 2017 | Goesan | 17G5A | Female | + |  |  | ○ |  |
| 2017 | Muan | 17M3A | Female | + |  |  | ○ |  |
| 2017 | Muan | 17M4A | Male | + |  |  |  | ○ |
| 2017 | Miryang | 17R3A | Female | + |  |  |  | ○ |
| 2018 | Paju | 18P1A | Male | + |  |  | ○ |  |
| 2018 | Paju | 18P3A | Male | - |  |  |  |  |
| 2018 | Paju | 18P3B | Male | + |  |  |  | ○ |
| 2018 | Paju | 18P3C | Female | + |  |  |  | ○ |
| 2018 | Paju | 18P3D | Female | + |  |  |  | ○ |
| 2018 | Paju | 18P4A | Male | + |  |  |  | ○ |
| 2018 | Inje | 18I5A | Female | + |  |  |  | ○ |
| 2018 | Goesan | 18G1A | Female | + |  | ○ |  |  |
| 2018 | Gongju | 18J1A | Female | + |  |  |  | ○ |
| 2018 | Muan | 18M2A | Female | + |  |  |  | ○ |
| 2018 | Muan | 18M3A | Male | - |  |  |  |  |
| 2018 | Muan | 18M3B | Female | + |  |  | ○ |  |
| 2018 | Muan | 18M4A | Female | + |  |  |  | ○ |
| 2018 | Muan | 18M5A | Male | + |  |  | ○ |  |
| 2018 | Muan | 18M5B | Male | + |  |  |  | ○ |
| 2018 | Muan | 18M5C | Male | + |  |  |  | ○ |
| 2018 | Andong | 18A1A | Female | + |  |  |  | ○ |
| 2018 | Miryang | 18R5A | Male | + |  |  |  | ○ |

**S1 Table.** **Infection prevalence and clade composition of the genus *Burkholderia* in the overwintering *R. pedestris* detected over 2-yr field survey.**
