# Supplementary material for: Abundance and diversity of gut-symbiotic bacteria, the genus Burkholderia in overwintering Riptortus pedestris (Hemiptera: Alydidae) populations and soil in South Korea
Source: PLoS One. 2019 Jun 13;14(6):e0218240. doi: 10.1371/journal.pone.0218240 (PMC6563995; doi:10.1371/journal.pone.0218240)
Supplement: S2 Table — (DOCX) [file pone.0218240.s002.docx]

**S2 Table.** ***Burkholderia* positive ratio and clade composition in soil samples collected over the two-year survey.**

| Year | Site | ID | *Burkholderia* infection | Clade composition | | | |
| --- | --- | --- | --- | --- | --- | --- | --- |
|  |  |  |  | PBE | BCC&P | SBE | Unclassified |
| 2017 | Paju | 17PJS1 | - |  |  |  |  |
| 2017 | Paju | 17PJS2 | + | ○ | ○ | ○ |  |
| 2017 | Paju | 17PJS3 | + | ○ | ○ | ○ |  |
| 2017 | Paju | 17PJS4 | - |  |  |  |  |
| 2017 | Paju | 17PJS5 | + | ○ | ○ | ○ |  |
| 2017 | Inje | 17IJS1 | - |  |  |  |  |
| 2017 | Inje | 17IJS2 | + | ○ |  |  |  |
| 2017 | Inje | 17IJS3 | + | ○ |  |  |  |
| 2017 | Inje | 17IJS4 | + | ○ |  |  |  |
| 2017 | Inje | 17IJS5 | + | ○ |  |  |  |
| 2017 | Gaesan | 17GSS1 | + | ○ | ○ |  |  |
| 2017 | Gaesan | 17GSS2 | + | ○ | ○ | ○ |  |
| 2017 | Gaesan | 17GSS3 | + | ○ | ○ |  |  |
| 2017 | Gaesan | 17GSS4 | + | ○ | ○ |  |  |
| 2017 | Gaesan | 17GSS5 | + | ○ | ○ |  |  |
| 2017 | Gongju | 17GJS1 | + |  |  |  | ○ |
| 2017 | Gongju | 17GJS2 | + |  |  |  | ○ |
| 2017 | Gongju | 17GJS3 | - |  |  |  |  |
| 2017 | Gongju | 17GJS4 | - |  |  |  |  |
| 2017 | Gongju | 17GJS5 | + | ○ |  |  |  |
| 2017 | Gochang | 17GCS1 | - |  |  |  |  |
| 2017 | Gochang | 17GCS2 | - |  |  |  |  |
| 2017 | Gochang | 17GCS3 | - |  |  |  |  |
| 2017 | Gochang | 17GCS4 | + | ○ |  |  |  |
| 2017 | Gochang | 17GCS5 | - |  |  |  |  |
| 2017 | Muan | 17MAS1 | + | ○ |  |  |  |
| 2017 | Muan | 17MAS2 | + | ○ |  |  |  |
| 2017 | Muan | 17MAS3 | - |  |  |  |  |
| 2017 | Muan | 17MAS4 | + | ○ |  |  |  |
| 2017 | Muan | 17MAS5 | - |  |  |  |  |
| 2017 | Miryang | 17MYS1 | + | ○ | ○ | ○ |  |
| 2017 | Miryang | 17MYS2 | + | ○ |  |  |  |
| 2017 | Miryang | 17MYS3 | + | ○ |  | ○ |  |
| 2017 | Miryang | 17MYS4 | - |  |  |  |  |
| 2017 | Miryang | 17MYS5 | + | ○ | ○ | ○ |  |
| 2017 | Andong | 17ADS1 | + | ○ | ○ | ○ |  |
| 2017 | Andong | 17ADS2 | - |  |  |  |  |
| 2017 | Andong | 17ADS3 | - |  |  |  |  |
| 2017 | Andong | 17ADS4 | - |  |  |  |  |
| 2017 | Andong | 17ADS5 | + | ○ | ○ | ○ |  |
| 2018 | Paju | 18PJS1 | + | ○ | ○ |  |  |
| 2018 | Paju | 18PJS2 | + | ○ | ○ |  |  |
| 2018 | Paju | 18PJS3 | + | ○ | ○ | ○ |  |
| 2018 | Paju | 18PJS4 | - |  |  |  |  |
| 2018 | Paju | 18PJS5 | + | ○ |  |  |  |
| 2018 | Inje | 18IJS1 | + | ○ | ○ | ○ |  |
| 2018 | Inje | 18IJS2 | + | ○ | ○ |  |  |
| 2018 | Inje | 18IJS3 | - |  |  |  |  |
| 2018 | Inje | 18IJS4 | - |  |  |  |  |
| 2018 | Inje | 18IJS5 | + |  |  |  | ○ |
| 2018 | Gaesan | 18GSS1 | + | ○ |  | ○ |  |
| 2018 | Gaesan | 18GSS2 | + | ○ | ○ |  |  |
| 2018 | Gaesan | 18GSS3 | + | ○ | ○ |  |  |
| 2018 | Gaesan | 18GSS4 | + | ○ | ○ |  |  |
| 2018 | Gaesan | 18GSS5 | + | ○ | ○ |  |  |
| 2018 | Gongju | 18GJS1 | - |  |  |  |  |
| 2018 | Gongju | 18GJS2 | - |  |  |  |  |
| 2018 | Gongju | 18GJS3 | + | ○ |  | ○ |  |
| 2018 | Gongju | 18GJS4 | + | ○ |  |  |  |
| 2018 | Gongju | 18GJS5 | - |  |  |  |  |
| 2018 | Gochang | 18GCS1 | - |  |  |  |  |
| 2018 | Gochang | 18GCS2 | - |  |  |  |  |
| 2018 | Gochang | 18GCS3 | - |  |  |  |  |
| 2018 | Gochang | 18GCS4 | - |  |  |  |  |
| 2018 | Gochang | 18GCS5 | + | ○ |  |  |  |
| 2018 | Muan | 18MAS1 | - |  |  |  |  |
| 2018 | Muan | 18MAS2 | + | ○ | ○ |  |  |
| 2018 | Muan | 18MAS3 | - |  |  |  |  |
| 2018 | Muan | 18MAS4 | - |  |  |  |  |
| 2018 | Muan | 18MAS5 | - |  |  |  |  |
| 2018 | Miryang | 18MYS1 | + |  |  |  | ○ |
| 2018 | Miryang | 18MYS2 | + | ○ | ○ |  |  |
| 2018 | Miryang | 18MYS3 | + | ○ | ○ |  |  |
| 2018 | Miryang | 18MYS4 | - |  |  |  |  |
| 2018 | Miryang | 18MYS5 | + | ○ | ○ | ○ |  |
| 2018 | Andong | 18ADS1 | - |  |  |  |  |
| 2018 | Andong | 18ADS2 | - |  |  |  |  |
| 2018 | Andong | 18ADS3 | - |  |  |  |  |
| 2018 | Andong | 18ADS4 | - |  |  |  |  |
| 2018 | Andong | 18ADS5 | - |  |  |  |  |
